# Genomic Detection of the Emerging, Highly Pathogenic HIV-1 Subtype D in Bahia, Northeast Brazil

**DOI:** 10.3390/v15081650

**Published:** 2023-07-29

**Authors:** Filipe Ferreira de Almeida Rego, Laise de Moraes, Marta Giovanetti, José Adriano Góes Silva, Felipe Guimarães Torres, Marcio de Oliveira Silva, Maria da Purificação Pereira da Silva, Johan Van Weyenbergh, Luciane Amorim Santos, Ricardo Khouri

**Affiliations:** 1 Escola Bahiana de Medicina e Saúde Pública, Avenida Dom João VI, 275, Brotas, Salvador 40290-000, Bahia, Brazil; filipefar@hotmail.com (F.F.d.A.R.); luciane.tika@gmail.com (L.A.S.); 2 Instituto Gonçalo Moniz, Fundação Oswaldo Cruz, Rua Waldemar Falcão, 121, Candeal, Salvador 40296-710, Bahia, Brazil; laisepaixao@live.com (L.d.M.); adrianofar@gmail.com (J.A.G.S.); fgtorres18@gmail.com (F.G.T.); 3 Programa de Pós-Graduação em Ciências da Saúde, Faculdade de Medicina da Bahia, Universidade Federal da Bahia, Praça Ramos de Queirós, s/n, Largo do Terreiro de Jesus, Salvador 40026-010, Bahia, Brazil; 4 Instituto Rene Rachou, Fundação Oswaldo Cruz, Avenida Augusto de Lima, 1715, Barro Preto, Belo Horizonte 30190-002, Minas Gerais, Brazil; giovanetti.marta@gmail.com; 5 Sciences and Technologies for Sustainable Development and One Health, University of Campus Bio-Medico, 21-00128 Rome, Italy; 6 Centro Estadual Especializado em Diagnóstico, Assistência e Pesquisa, Secretaria de Saúde do Estado da Bahia, Salvador 40110-050, Bahia, Brazil; oliveiras_m@yahoo.com.br (M.d.O.S.); mpuri@oi.com.br (M.d.P.P.d.S.); 7 Rega Institute for Medical Research, Department of Immunology, Microbiology and Transplantation, KU Leuven, Herestraat 49-Box 1030, 3000 Leuven, Belgium; j.vw@live.be

**Keywords:** HIV-1 subtype D, phylodynamics, genomic surveillance

## Abstract

(1) Background: The HIV subtype D is generally associated with a faster decline in CD4^+^ T cell counts, a higher viral load, and a faster progression to AIDS. However, it is still poorly characterized in Brazil. In this study, we used genomics and epidemiological data to investigate the transmission dynamics of HIV subtype D in the state of Bahia, Northeast Brazil. (2) Methods: To achieve this goal, we obtained four novel HIV-1 subtype D partial *pol* genome sequences using the Sanger method. To understand the emergence of this novel subtype in the state of Bahia, we used phylodynamic analysis on a dataset comprising 3704 *pol* genome sequences downloaded from the Los Alamos database. (3) Results: Our analysis revealed three branching patterns, indicating multiple introductions of the HIV-1 subtype D in Brazil from the late 1980s to the late 2000s and a single introduction event in the state of Bahia. Our data further suggest that these introductions most likely originated from European, Eastern African, Western African, and Southern African countries. (4) Conclusion: Understanding the distribution of HIV-1 viral strains and their temporal dynamics is crucial for monitoring the real-time evolution of circulating subtypes and recombinant forms, as well as for designing novel diagnostic and vaccination strategies. We advocate for a shift to active surveillance, to ensure adequate preparedness for future epidemics mediated by emerging viral strains.

## 1. Introduction

The human immunodeficiency virus type 1 (HIV-1), the etiological agent of acquired immunodeficiency syndrome (AIDS), infects around 38,4 million people worldwide. It presents a highly diverse genome of approximately 9.5 kb in length, formed by two single RNA strands. Its genetic diversity can be classified in a wide variety of groups (M, N, O, and P). The HIV-1 group M viruses can be further subdivided into subtypes (A1, A2, A3, A4, A6, A7, A8, B, C, D, F1, F2, G, H, J, and K), unique recombinant (URF), and circulating (CRF) forms [1].

Since the 1990s, molecular epidemiological studies of HIV-1 in Brazil have aimed to identify and understand the distribution of different subtypes and recombinant forms throughout the country [2,3,4]. In the past decade, other studies have shed light on the dissemination, incidence of primary resistance mutation, and origin of several HIV-1 subtypes, particularly B and C, in Brazil [5,6,7,8,9,10]. Such studies play a vital role in conducting HIV-1 genomic surveillance in Brazil and have the potential to uncover the emergence of more pathogenic variants within the country, similar to previous findings of the CRF19 recombinant in Cuba and the recently described “VB” variant in the Netherlands [11,12].

The prevalence of different viral subtypes in Brazil varies by region, with the most common subtypes being B, F, C, and the recombinant form BF [13]. In Northeast Brazil, the prevalence of subtypes B, F, C, and BF recombinants is 76%, 8%, 2%, and 7%, respectively [14]. A similar distribution of subtypes is observed in Bahia, with subtype B being the first to be identified in the state. The estimated prevalence for subtypes B, F, C, and BF recombinants ranges from 67.2% to 91.8%, 1.8% to 14.4%, 1.7% to 4.1%, and 3.3% to 24.1%, respectively [15,16,17,18,19].

Despite the high frequency of those subtypes, other viral strains have already been described in Brazil, including subtype D, which has been suggested to be more pathogenic than other forms [20,21,22,23,24,25]. However, despite the importance of the HIV-1 subtype D surveillance, there is still a paucity of studies in Brazil that describe its phylogenetic relationship with sequences from other countries and its transmission dynamics within Brazilian regions.

In this study, we provide insights into the spread of subtype D in the state of Bahia by sequencing the *pol* region of the first four HIV-1 positive patients belonging to this subtype. To gain a comprehensive understanding of subtype D’s dispersion in Brazil, we conducted a phylodynamic analysis using reference sequences from the Los Alamos database, including all available Brazilian strains. Our analysis revealed multiple introduction events of subtype D in Brazil from Europe and Africa and highlighted South Africa as the primary source driving its nationwide spread.

## 2. Materials and Methods

### 2.1. HIV-1 Samples from Bahia

A total of four subjects were diagnosed as HIV-1 subtype D positive between 2014 and 2015 and received follow up at the Specialized Center for Diagnosis, Care, and Research (CEDAP), a state government public health reference service located in the city of Salvador, Bahia, Northeast Brazil. This study was conducted in accordance with the Declaration of Helsinki and was approved by the Institutional Review Board of the Instituto Gonçalo Moniz (IGM-FIOCRUZ) (protocol number 1.764.505).

### 2.2. HIV-1 Sequencing, Assembly, and Subtyping

The viral RNA isolation was performed using a QIAamp Viral RNA Mini Kit (Qiagen, Germany) according to the manufacturer’s instructions. The protease/reverse transcriptase (PR/RT) region was amplified and sequenced as previously described [26]. The outer polymerase chain reaction (PCR) was performed using a SuperScript III One-Step RT-PCR System with Platinum Taq DNA Polymerase (Thermo Fisher Scientific, United States of America) and the following primers: K1 (CAGAGCCAACAGCCCCACC) and K2 (TTTCCCCACTAACTTCTGTATGTCATTGACA) [27]. Inner PCR was performed using Platinum Taq DNA Polymerase High Fidelity (Thermo Fisher Scientific, United States of America) and with the following primers: DP16 (CCTCAAATCACTCTTTGGCAAC) and RT4 (AGTTCATAACCCATCCAAAG) [28]. The generated inner PCR products were then sequenced using ABI 3500xL Genetic Analyzer (Applied Biosystems, United States of America) with the following primers: F1 (GTTGACTCAGATTGGTTGCAC), F2 (GTATGTCATTGACAGTCCAGC) [29], DP10 (CAACTCCCTCTCAGAAGCAGGAGCCG), DP11 (CCATTCCTGGCTTTAATTTTACTGGTA) [30], RT4 (AGTTCATAACCCATCCAAAG), GABO1 (CTCARGACTTYTGGGAAGTTC), and GABO2 (GCATCHCCCACATCYAGTACTG) [26].

Sequence visualization, editing, and assembly were performed using Geneious v.10.0.8 software [31]. Subtyping was determined using the REGA HIV-1 Subtyping Tool v.3.46 available at Genome Detective (https://www.genomedetective.com) accessed on 5 March 2023, and the jpHMM (jumping profile Hidden Markov Model), which is a probabilistic generalization of the jumping-alignment approach [32].

### 2.3. HIV-1 Pol Reference Sequences from the Los Alamos Database

To perform phylodynamic and phylogenetics analyses, HIV-1 subtype D *pol* genome sequences over 900 base pairs covering the protease and reverse transcriptase (starting from genomic position 2256 and ending at position 3234 relative to the HXB2 reference), along with the sample collection date and location, were downloaded from Los Alamos HIV Sequence Database (https://www.hiv.lanl.gov) up to 15 July 2022. Sequences identified as duplicates or with 100% identity belonging to the same country and year were excluded. Additionally, we excluded possible recombinant sequences and sequences that do not belong to the analyzed genomic region of interest. Furthermore, sequences without a collection date or country, as well as Brazilian sequences without state information, were also excluded from the analysis.

### 2.4. Maximum Likelihood

Sequences were aligned using MAFFT v.7.455 [33,34] and manually edited using Geneious software [29]. Sequences that were too short (<900 base pair) or did not correspond to the analyzed region were excluded. The phylogenetic signal and the best fitting evolutionary model were evaluated using the software IQ-TREE v.2.0.3 [35]. A maximum likelihood (ML) tree was estimated using IQ-TREE v.2.0.3 [36] under GTR+F+I+G4 nucleotide substitution model [37,38] with 1000 replicates and an ultrafast bootstrap [39]. Bootstrap was considered significant when >90%. The ML trees were visualized using FigTree v.1.4.4 (http://tree.bio.ed.ac.uk/software/figtree/) accessed on 30 November 2022 and plotted using the ggtree package in RStudio v4.2.2 (https://www.r-project.org) accessed on 5 March 2022 [40].

### 2.5. Molecular Clock Phylogenetic Analysis

To determine the tMRCA (time to most recent common ancestor) of clades that include Brazilian sequences, a Bayesian analysis was performed [41]. The presence of a temporal signal was evaluated using TempEst v.1.5.3 [42]. Time-scaled phylogenetic trees were inferred using the BEAST v.1.10.4 package [41] with BEAGLE v4.0.0 to improve the computational performance [43,44]. We employed a stringent model selection analysis using both path-sampling (PS) and steppingstone (SS) procedures to estimate the most appropriate molecular clock model for the Bayesian phylogenetic analysis [45]. The uncorrelated relaxed molecular clock model was chosen for all datasets as indicated by estimating marginal likelihoods, also employing the codon-based SRD06 model of nucleotide substitution and the nonparametric Bayesian Skyline coalescent model. MCMC analyses were performed in BEAST v.1.10.4, running in duplicate for 300 million interactions and sampling every 30,000 steps in the chain [46,47]. Convergence for each run was assessed in Tracer (effective sample size for all relevant model parameters >200). MCC trees for each run were summarized using TreeAnnotator v.1.10.4 after discarding the initial 10% as burn-in. Posterior probability was considered significant when ≥0.9.

## 3. Results and Discussion

**Figure 1:**
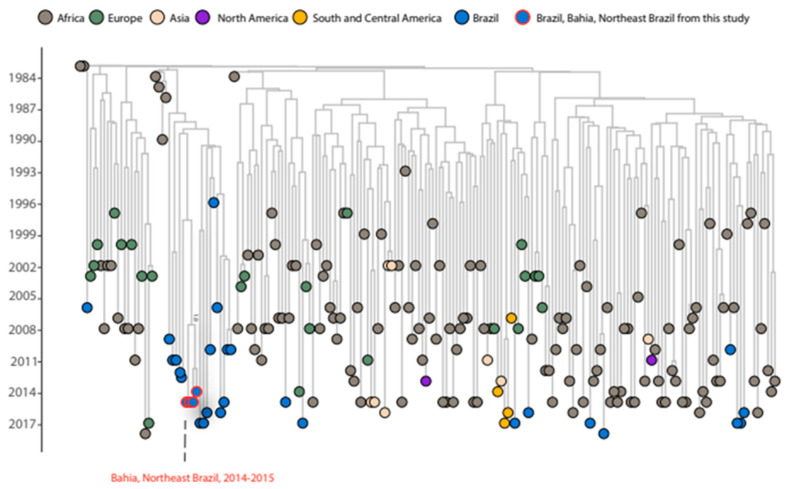
Bayesian phylogeny containing 203 HIV-1 subtype D sequences from pol region.

To understand the introduction and spread of HIV-1 subtype D in Brazil, a worldwide dataset was built. First, 3808 sequences of *pol* region were downloaded from Los Alamos database. Among them, 104 were excluded for not belonging to the analyzed region of interest. Of the remaining 3704 sequences, 1759 were excluded for being identical, or highly similar with other sequences belonging to the same collection date and location. This step was taken to improve the precision and efficiency of the analyses, particularly by excluding sequences with high similarity if they belonged to the same country. It is worth noting that these similar sequences were specifically from Uganda and led to multiple polytomies. The 1945 remaining sequences, which included 27 (1996–2018) previously published sequences from Brazil (eleven from Rio de Janeiro, four from Rio Grande do Sul, five from Pará, one from Goiás, two from São Paulo, and four not informed) and four newly identified sequences from the state of Bahia (2014–2015) (Table 1), were used to reconstruct the ML tree (Supplementary Figure S1). The clades that included Brazilian sequences were then extracted for the Bayesian analysis, resulting in 203 sequences being analyzed (Figure 1).

Our analysis revealed that the Brazilian sequences are distributed across nine distinct clades, suggesting multiple introductions events of this subtype in the country (Supplementary Figure S1A). The Bayesian analysis shows that the multiple introductions of HIV-1 subtype D in Brazil occurred at different times from the late 1980s to the late 2000s (Figure 1) and likely originated from Europe (Portugal, France, and Spain), Eastern Africa (Kenya, Congo, and the Democratic Republic of the Congo) and Western Africa (Senegal), and Southern Africa (South Africa).

One sequence collected in 2017 from São Paulo was found to be closely related to sequences from Portugal with a bootstrap value of 100% (Supplementary Figure S1E). This clade was estimated to have originated around 1999, with 95% Bayesian high posterior density (HPD) between 1993-01-10 and 2003-02-17 (pp = 0.99) (Figure 1). Two sequences isolated from Rio de Janeiro in 2016 and 2017 were grouped with two sequences from France and one from Spain with a statistical support of 90% (Supplementary Figure S1G). However, this clade did not have statistical support on the Bayesian tree (pp = 0.53). Nonetheless, the clade with the sequence isolated in 2016 sharing a common ancestor with sequences from France has a tMRCA of 1995 (95% HPD: 1991-07-31:2000-02-17, pp = 0.98) (Figure 1). These findings are similar to those of HIV-1 subtype C and F1, which also suggest that Europe was the source of the introduction of these viruses in Brazil [48,49,50].

A group of four sequences from Rio Grande do Sul collected between 2010 and 2017 were grouped with a sequence from Kenya (bootstrap value = 98%) (Supplementary Figure S1B). This group of sequences from Rio Grande do Sul and the sequence from Kenya shared a common ancestor around 1992 (95% HPD: 1988-01-11:1996-11-10, pp = 0.98) (Figure 1). One sequence isolated from Pará, a state located in the north region of Brazil, in 2018 clustered together with sequences from Uganda (bootstrap value = 100%). The tMRCA for this clade is 2008 (95 % HPD: 2005-03-07:2009-12-24, pp = 1) (Figure 1). The sequence from Goiás (Midwest region), collected in 2017 (bootstrap value = 100%), was clustered with two sequences from Uganda (Supplementary Figure S1D). Another sequence from Pará, collected in 2015, was clustered with sequences from the Democratic Republic of the Congo and Senegal (bootstrap value = 97%) (Supplementary Figure S1F). A Brazilian sequence from Rio de Janeiro, collected in 2006, also grouped with a sequence from the Democratic Republic of the Congo (bootstrap value = 100%) (Supplementary Figure S1H). These clades from Goiás, Pará, and Rio de Janeiro did not have statistical support for tMRCA inference, with pp values of 0.49, 0.02, and 0.11, respectively. Another sequence isolated in Pará in 2017 clustered together with sequences from Uganda but without statistical significance and was excluded from Bayesian inference (bootstrap value = 69%) (Supplementary Figure S1J). These findings are in accordance with studies that show the relationship between Brazilian and African sequences, especially in HIV-1 subtypes C and HIV-1 CRF02_AG [6,51].

The nineteen remaining Brazilian sequences, which correspond to 61% of total subtype D Brazilian sequences, including those sequenced in this study, were clustered in a monophyletic group with 100% bootstrap statistical support (Supplementary Figure S1I). This clade contains sequences from samples collected between 1996 to 2017 from different regions (Northeast, North, and Southeast) and shares a common ancestor around 1987 with statistical support (95% HPD: 1983-05-27:1990-07-31, pp = 0.99) (Figure 1). The four new sequences from Bahia were also grouped into a monophyletic cluster inside this Brazilian cluster, suggesting a single introduction of this virus in the state, sharing a common ancestor with a sequence from Pará with tMRCA of 1997 (95% HPD: 1990-03-04:2007-05-05, pp = 1) (Figure 1). No epidemiological relationship was observed among these sequences. Of note, this Brazilian clade, which contains 19 sequences, including the four new sequences from Bahia, was clustered with sequences isolated from South Africa between 1984 to 1990, sharing a common ancestor with an introduction date of 1983 with statistical support (95% HPD: 1983-08-15:1984-01-22, pp = 0.99); however, the absence of comprehensive genomic surveillance worldwide may affect these results.

Multiple introductions of HIV-1 subtype D in Brazil from different world regions, including Africa and Europe, have occurred at different times over the last few decades. These findings are consistent with other viral subtypes, where Africa is the epicenter of the HIV epidemic and the place of origin of the virus, and Europe serves as a transitory source for the passage of these viruses [6,48,52]. Although HIV-1 subtype D is more pathogenic and, therefore, should be considered a public health concern, this is the first study in Brazil that demonstrates the origin and dispersion dynamic of this subtype in the country, reporting the first *pol* sequences of this subtype from the Brazilian Northeast region. Our findings underscore the importance of enhancing genomic surveillance in Brazil and other countries, such as South Africa, to promptly detect and respond to viral outbreaks. However, the limited availability of complete HIV-1 genome sequences in these regions hampers our ability to assess the regional molecular epidemiology of viral strains. Furthermore, conducting a comprehensive analysis with a larger number of sequences is necessary to elucidate the dynamics of HIV-1 subtype D dispersion.

## Figures and Tables

**Table 1:** Clinical and demographic characteristics of four patients described in this study.

Patient ID	Gender	Age	Viral Load (Copies/mL)	CD4 Cell Count/m^3^	CD8 Cell Count/m^3^	CD4/CD8 Ratio	CD45 Cell Count/m^3^
HV0018	Female	29	216	1553	1399	1.11	4022
HV0206	Male	26	50,273	704	1231	0.57	2315
HV0220	Male	31	138,217	466	698	0.67	2263
HV0225 *	Male	53	NI	NI	NI	NI	NI

NI = not informed. * Patient did not return for follow up after diagnostic.

## Data Availability

The new sequences have been deposited in NCBI GenBank under accession numbers MW596909, MW596999, MW597006, and MW597008.

## Supplementary Materials

The following supporting information can be downloaded at: https://www.mdpi.com/article/10.3390/v15081650/s1, Figure S1: Phylogenetic tree of HIV-1 subtype D, branches with Brazilian sequences are highlighted: (A) Maximum likelihood tree of 1945 HIV-1 subtype D sequences; (B) Highlighted branch with Brazilian sequences from Rio Grande do Sul; (C) Highlighted branch with a Brazilian sequence from Pará; (D) Highlighted branch with a Brazilian sequence from Goiás; (E) Highlighted branch with a Brazilian sequence from São Paulo; (F) Highlighted branch with another Brazilian sequence from Pará; (G) Highlighted branch with Brazilian sequences from Rio de Janeiro; (H) Highlighted branch with a Brazilian sequence from Rio de Janeiro; (I) Highlighted branch of the major Brazilian clade; (J) Highlighted branch with another Brazilian sequence from Pará.
