# Supplementary material for: Genomic Detection of the Emerging, Highly Pathogenic HIV-1 Subtype D in Bahia, Northeast Brazil
Source: Viruses. 2023 Jul 29;15(8):1650. doi: 10.3390/v15081650 (PMC10458544; doi:10.3390/v15081650)
Supplement: Supplementary file 1 [file viruses-2454914-supplementary.pdf › viruses-2454914-supplementary.pdf]

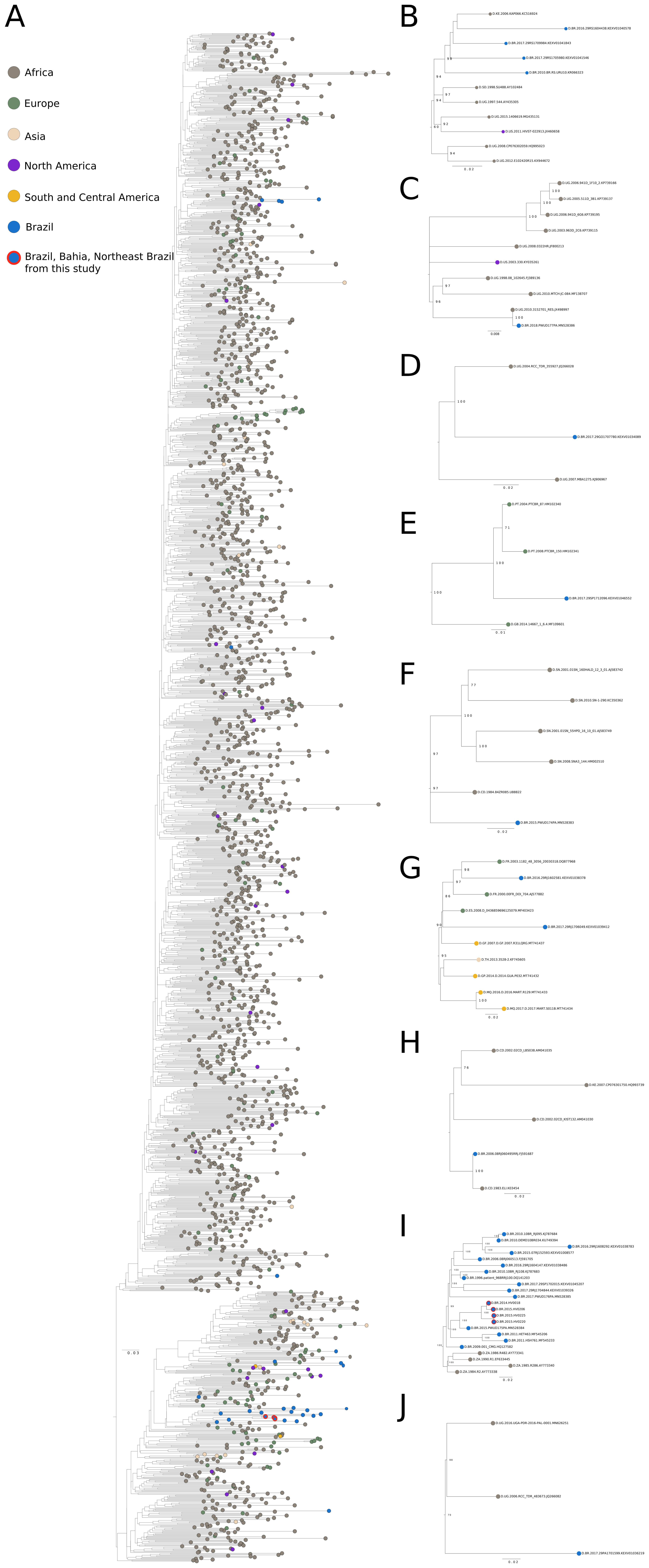
A
Africa
Europe
Asia
North America
South and Central America
Brazil
Brazil, Bahia, Northeast Brazil from this study
0.03
B
C
D
E
F
G
H
I
J
